# Hypoxic regulation of cytoglobin and neuroglobin expression in human normal and tumor tissues

**DOI:** 10.1186/1475-2867-10-33

**Published:** 2010-09-09

**Authors:** Marwan Emara, A Robert Turner, Joan Allalunis-Turner

**Affiliations:** 1 Department of Oncology, University of Alberta, Cross Cancer Institute, 11560 University of Alberta, Edmonton, Alberta, T6G 1Z2, Canada

## Abstract

**Background:**

Cytoglobin (Cygb) and neuroglobin (Ngb) are recently identified globin molecules that are expressed in vertebrate tissues. Upregulation of Cygb and Ngb under hypoxic and/or ischemic conditions *in vitro *and *in vivo *increases cell survival, suggesting possible protective roles through prevention of oxidative damage. We have previously shown that Ngb is expressed in human glioblastoma multiforme (GBM) cell lines, and that expression of its transcript and protein can be significantly increased after exposure to physiologically relevant levels of hypoxia. In this study, we extended this work to determine whether Cygb is also expressed in GBM cells, and whether its expression is enhanced under hypoxic conditions. We also compared Cygb and Ngb expression in human primary tumor specimens, including brain tumors, as well as in human normal tissues. Immunoreactivity of carbonic anhydrase IX (CA IX), a hypoxia-inducible metalloenzyme that catalyzes the hydration of CO_2 _to bicarbonate, was used as an endogenous marker of hypoxia.

**Results:**

Cygb transcript and protein were expressed in human GBM cells, and this expression was significantly increased in most cells following 48 h incubation under hypoxia. We also showed that Cygb and Ngb are expressed in both normal tissues and human primary cancers, including GBM. Among normal tissues, Cygb and Ngb expression was restricted to distinct cell types and was especially prominent in ductal cells. Additionally, certain normal organs (*e.g. *stomach fundus, small bowel) showed distinct regional co-localization of Ngb, Cygb and CA IX. In most tumors, Ngb immunoreactivity was significantly greater than that of Cygb. In keeping with previous *in vitro *results, tumor regions that were positively stained for CA IX were also positive for Ngb and Cygb, suggesting that hypoxic upregulation of Ngb and Cygb also occurs *in vivo*.

**Conclusions:**

Our finding of hypoxic up-regulation of Cygb/Ngb in GBM cell lines and human tumor tissues suggests that these globin molecules may be part of the repertoire of defense mechanisms that allow cancer cells to survive in hypoxic microenvironments.

## Background

A third member of the vertebrate globin family, neuroglobin (Ngb), was discovered in 2000 and so-named because it is primarily expressed in neuronal tissue, including retina [1]. Shortly thereafter, a fourth vertebrate globin--cytoglobin (Cygb), was described independently by three groups [2-4]. Cygb is expressed ubiquitously in human tissue [2], however, low cellular levels of Cygb and Ngb (μM range) may have impeded their earlier discovery [5]. The amino acid sequences of Cygb and Ngb show little similarity to that of hemoglobin (Hb) or myoglobin (Mb) (< 30% and <25% identity for Cygb and Ngb, respectively). However, amino acids that confer Hb and Mb function are conserved together with all features of the globin fold [2,4,6]. Unlike Hb and Mb, the physiological roles of Ngb and Cygb are incompletely understood and several functions are conceivable. Ngb and Cygb may function as a Mb-type molecule to store O_2 _thus facilitating O_2 _diffusion to mitochondria [1,6,7]. However, the lower O_2 _affinity of Ngb (*P*_50 _of 7.5 torr under physiological conditions of pH and temperature) [8] compared to that of Mb (*P*_50 _of 2-3 torr) [7] does not support an O_2 _storage function for Ngb in neuronal tissue, including retina, as only a small fraction of Ngb (~ 12%) will be O_2 _saturated under normal conditions [5,8-10].

Similar to Mb, but in contrast to Ngb, the O_2 _binding of Cygb is pH-independent [8] with higher O_2 _affinity values (*P*_50 _of 0.7-1.8 torr) [4,8,11], thus suggesting a possible physiological role to supply O_2_. However, due to its low concentration *in vivo*, Cygb function may be restricted to O_2_-requiring cellular reactions unrelated to mitochondrial respiration [8].

In brain, Ngb is upregulated under hypoxic/ischemic conditions [12] and may function to scavenge reactive oxygen (ROS) and nitrogen species (RNS) that are a major cause of cellular damage [12,13]. It has been shown that in the Fe^2+^-NO form, Ngb reacts more rapidly with peroxynitrite than does Hb [14]. Additionally, in contrast to Hb and Mb, the reaction of met(Fe^3+^)Ngb with peroxynitrite or hydrogen peroxide does not appear to generate the cytotoxic ferryl (Fe^4+^) species, and this may contribute to cellular survival [5,14]. However, evidence for Ngb's neuroprotective function *in vivo *is inconsistent [10,12,13,15-17]. Similarly, Cygb was found to be upregulated following oxidative stress and hypoxic/ischemic conditions *in vitro *and *in vivo *[17-21], and overexpression of Cygb in hepatic stellate cells, human SH-SY5Y neuroblastoma cells and MIN6 cells is protective [17,22,23]. The function of Cygb has not yet been intensively investigated. In fibroblasts and related cells expressing Cygb, its expression has been linked to collagen production and organ fibrosis [24-27]. Recently, it has been proposed that CYGB may function as a tumor suppressor gene as hypermethylation of its promoter was detected in primary human non-small cell lung cancers (48%) [28] and oral cancers (65%) [29], and in lung (8 of 10) and breast (4 of 4) cancer cell lines [30].

Glioblastoma multiforme (GBM) is the most common brain tumor among adults comprising 25% of all malignant nervous system tumors. Its resistance to multimodality therapy confers a poor prognosis and the 2-year survival rate remains only 10-25% [31]. Necrosis, and by inference hypoxia, is a diagnostic feature of human GBM tumors [32]. Hypoxic microenvironments frequently occur in human tumors, and tumor cells that are hypoxic are resistant to ionizing radiation and certain chemotherapeutic agents, are genetically unstable and metastasize frequently. Further, hypoxic microenvironments also select for tumor cells with reduced apoptotic potential. The presence of tumor hypoxia is an indicator of poor prognosis for both local-regional control and progression-free survival, and there is also evidence that hypoxia *per se *selects for a more aggressive tumor phenotype (reviewed in [33] and references therein). Tumor cells that survive in hypoxic microenvironments must first sense changes in [O_2_] and then activate defense and adaptation mechanisms [34]. Previous work suggests that increased Ngb expression may be part of the repertoire of hypoxia defense mechanisms in normal brain. Although some studies have reported that Ngb is expressed exclusively in neurons but not in glia [35,36], others have reported Ngb expression in astrocytes cultured from newborn mouse brain [37], and we have shown that Ngb is expressed and upregulated by hypoxia in human GBM cell lines [38]. Here, we examined whether Cygb is also expressed in human GBM cell lines and tested whether physiologically relevant levels of hypoxia can modulate its expression as was previously demonstrated for Ngb. In addition to these *in vitro *analyses, we used human tissue microarrays (TMA) to assess whether Ngb/Cygb is more broadly expressed among human primary tumors, including brain cancers, and their adjacent normal tissues. CA IX immunoreactivity was used as an endogenous marker for tissue hypoxia.

## Results

### Cygb transcript and protein are expressed in GBM cells *in vitro*

**Figure 1:**
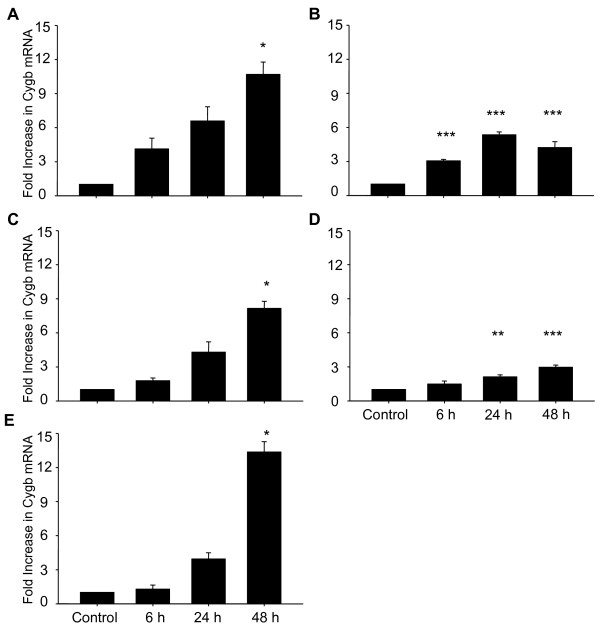
**Cygb mRNA expression in human GBM cells**. Cygb mRNA expression was assessed by qRT-PCR after exposure to hypoxia (0.6% O_2_) for 0, 6, 24 and 48 h. Data were expressed as fold increase relative to aerobic control (n = 4). (A) M006x: (B) M006xLo; (C) M010B; (D) M059J; (E) U87T. *p < 0.05; **p < 0.01; ***p < 0.001 (ANOVA).

**Figure 2:**
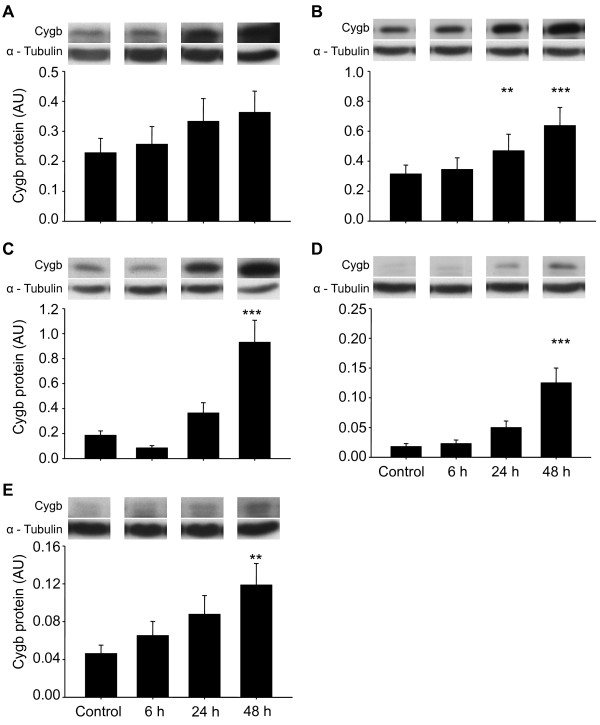
**Cygb protein expression in human GBM cells**. Cygb expression was assessed by Western blot analyses after exposure to hypoxia (0.6% O_2_) for 0, 6, 24 and 48 h (n = 4). The integrated intensities of Cygb and α- tubulin (control) bands were determined and expressed in arbitrary units (AU), and representative blots are shown. (A) M006x: (B) M006xLo; (C) M010B; (D) M059J; (E) U87T. *p < 0.05; **p < 0.01; ***p < 0.001 (ANOVA).

Cygb mRNA was detected in all aerobic GBM cell lines (controls), and when cultured under hypoxic conditions (0.6% O_2 _× 48 h), all cell lines showed significantly increased expression of Cygb mRNA (M006x, M010b and U87T, p < 0.05; M006xLo and M059J, p < 0.001) (Figure 1). Two cell lines also showed significant increases in Cygb transcript at earlier time points (M006xLo at 6 and 24 h, p < 0.001; M059J at 24 h, p < 0.01). Consistent with qRT-PCR results, Cygb protein was detected in all aerobic GBM cell lines with hypoxia-tolerant cells (M006x, M006xLo) showing the highest basal levels of Cygb. After 48 h of hypoxia, Cygb protein was significantly increased in four of five cell lines (M006xLo, M010B, and M059J, p < 0.001; U87 T, p < 0.01), with the greatest relative increases seen in hypoxia-sensitive cells M010b and M059J (Figure 2). Cygb protein was also significantly increased in M006xLo cells after 24 h of hypoxia (p < 0.01). M006x cells showed a modest increase in Cygb protein after hypoxia, but this was not significant (Figure 2A). There was no correlation between the magnitude of Cygb protein increase after hypoxia and the respective basal levels of each cell line.

### Cygb and Ngb are expressed in human normal tissues and cancers

**Figure 3:**
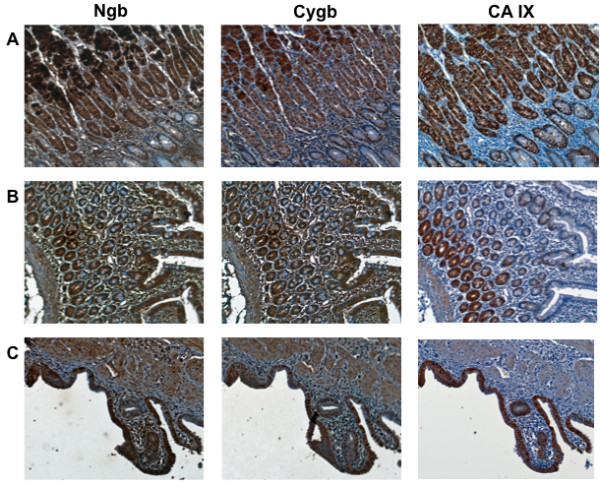
**Expression of Ngb, Cygb and CA IX in human normal tissues**. Tissue microarrays containing cores obtained from various human normal tissues were stained with to antibodies to Ngb, Cygb or CA IX. Positive staining was visualized by the chromogenic reaction of HRP with DAB. Photomicrographs were obtained at 20× magnification, and the scale bar indicates 50 μM. (A) stomach (fundus); (B) small bowel; (C) gallbladder.

Immunohistochemical staining of TMAs showed that Ngb and Cygb were variably expressed in both normal tissues and tumors. As reported previously [39], Ngb was found primarily in the cytoplasm, whereas Cygb was detected in both nucleus and cytoplasm. Among normal tissues, Ngb expression was greatest in bone, liver, sigmoid colon, rectum and kidney, with low or negligible levels in skin, muscle and lung (Table 1). Tissues with highest Cygb expression were stomach fundus, kidney tubules and cerebellum. However, compared to Ngb, Cygb levels were reduced overall and were low or absent in approximately half of the tissues. There was no significant correlation between levels of Cygb and Ngb protein in these normal tissues (p < 0.07). However, two features of Cygb and Ngb expression in normal tissues were of particular interest. First, in several normal tissues, expression of Cygb and Ngb was not uniform throughout the tissue. Rather, distinct cell types or tissue structures frequently showed strong positive staining while the remainder of the tissue showed weak or absent staining. Among the structures strongly stained for Cygb were ductal cells of the breast and kidney, secretory cells of the salivary gland, white pulp/lymph of the spleen, and tips of desquamating cells found in several types of normal tissue. Ngb staining was also prominent among ductal cells of breast, endometrium, testis, prostate and salivary gland. Both Ngb and Cygb were generally absent from stroma. Second, most normal tissues showed low or absent CA IX immunoreactivity. However, stomach, small bowel, and gallbladder showed nearly identical patterns of Ngb, Cygb and CA IX staining (Figure 3).

**Table 1:** Ngb, Cygb and CA IX expression in tissue microarrays of human solid tumors and adjacent normal tissues

Organ	NGB	CGB	CAIX	Comments
***Normal***				
Skin	1	1	0	Cygb: + desquamating cells
Breast	1	1	0	Ngb & Cygb: + ductal cells
Spleen	2	1	0	
Lymph node	3	1	1	Ngb & Cygb: + white pulp, lymph
Skeletal muscle	0	0	0	
Lung	0	0	0	
Salivary gland	2	2	1	Ngb & Cygb: + ductal cells
Liver	3	2	1	
Gallbladder	2	1	1	
Pancreas	3	0	0	
Tonsil	2	1	1	
Esophagus	2	0	1	
Stomach, antrum	2	0	1	Ngb & Cygb: + ductal cells
Stomach, fundus	3	2	2	Ngb & Cygb: + ductal cells
Small bowel	3	1	2	Ngb & Cygb: + ductal cells
Kidney, cortex	3	2	1	Ngb & Cygb: + collecting ducts & tubules; - glomeruli
Kidney, medulla	3	2	1	Ngb & Cygb: + collecting ducts & tubules; - glomeruli
Urinary bladder	3	1	1	
Prostate	4	1	1	
Testis	1	0	0	Ngb & Cygb: + ductal cells
Uterine cervix	1	1	0	Ngb & Cygb: + desquamating cells, - basal layer
Endometrium	2	0	0	Ngb & Cygb: + ductal cells
Myometrium	2	1	1	
Placenta	1	0	0	Ngb & Cygb: + ductal cells
Adrenal gland	*NE*	2	0	Cygb: + ductal cells
Thyroid	*NE*	*NE*	*NE*	
Cerebrum	3	3	1	
Cerebellum	2	2	1	
***Tumor***				
Skin	1	1	1	
Skin	*NE*	*NE*	*NE*	Ngb & Cygb: indistinguishable from melanin
Subcutis	1	0	0	
Breast	4	1	0	Ngb & Cygb: + ductal cells
Breast	3	1	2	Ngb & Cygb: + ductal cells
Hodgkins				
lymphoma	3	2	0	
Bone	5	0	0	
Lung	3	0	0	
Lung	2	1	1	
Liver	4	2	0	
Liver	4	3	2	
Liver	5	2	2	
Esophagus	2	0	1	
Stomach	1		0	
Stomach	3		1	
Stomach	3		0	
Duodenum	1	??	0	
Sigmoid colon	4		0	
Rectum	4		1	Ngb: + ductal cells
Kidney	4		0	Ngb: + villi, - stroma; Cygb:+ normal & tumor cells
Urinary bladder	1		3	Ngb: + infiltrating cells
Prostate	3		1	Ngb: + ductal cells
Testis	2		0	Ngb: + ductal cells
Uterine cervix	2		1	
Endometrium	1		1	Ngb: some + ductal cells
Ovary	2		0	
Ovary	3		0	
Ovary	1		1	
Thyroid	2		3	

Tissue microarrays constructed from cores obtained from various human solid tumors and normal adjacent tissues were purchased from IMGENEX Corporation (San Diego, CA). Immunopositive staining for Ngb, Cygb and CA IX was assessed by two observers who noted the overall tissue staining and the presence of positively stained focal regions. Each section was assigned an overall score, where 0 = absence of positively stained cells; 1 = a few positive cells/regions ( < 10%); 2 = weak positive staining, occasional positive foci; 3 = intermediate staining, occasional positive foci ( < 50%); 4 = strongly positive in most of the section, several positive foci; and 5 = strongly positive throughout with many intensely positive foci. Distinct features of the immunostaining patterns in individual tissue cores are noted in the comments column, where "+" = positive staining, "-" = negative staining and *NE *= not evaluable.

**Figure 4:**
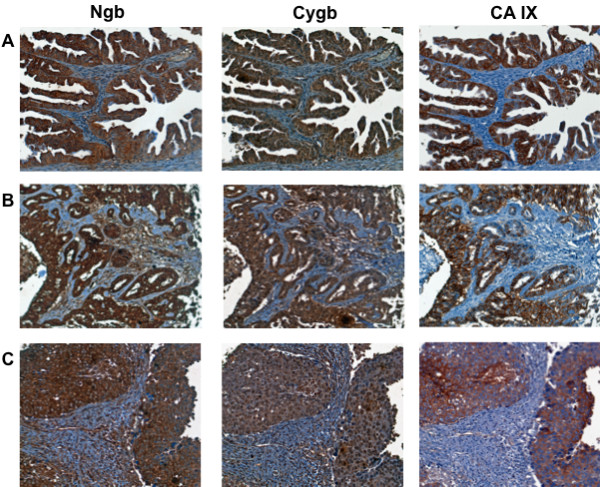
**Expression of Ngb, Cygb and CA IX in human tumors**. Tissue microarrays containing cores obtained from various human tumors were stained with to antibodies to Ngb, Cygb or CA IX. Positive staining was visualized by the chromogenic reaction of HRP with DAB. Photomicrographs were obtained at 20× magnification, and the scale bar indicates 50 μM. (A) ovarian carcinoma; (B) hepatocellular carcinoma; (C) breast infiltrating duct carcinoma.

Tumor sections showed three distinct staining patterns for Cygb/Ngb: (1) a uniform expression throughout the non-stromal tissue; (2) distinct regions of positively staining cells among otherwise negative or weakly staining tissue; or (3) densely staining focal areas frequently including intensely positive foci. Among individual tumor sections included in the 'tumor/matched normal tissue' TMA, Ngb and Cygb expression was generally increased compared to levels observed in corresponding normal tissues. As well, Cygb and Ngb immunoreactivity showed similar patterns of distribution. Most tumor sections (24/29) showed low or absent CA IX immunoreactivity. However, the breast, liver, bladder and thyroid tumors that contained regions strongly positive for CA IX also showed in matching sections similar staining patterns for Cygb and Ngb (Figure 4).

### Cygb and Ngb are expressed in human brain tumors

**Figure 5:**
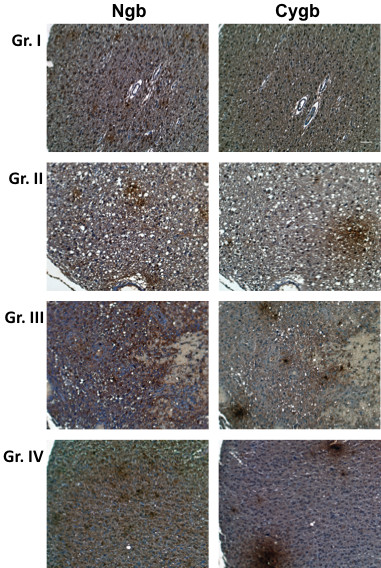
**Expression of Ngb and Cygb in human astrocytomas**. Tissue microarrays containing cores obtained from various human brain tumors were stained with to antibodies to Ngb or Cygb. Positive staining was visualized by the chromogenic reaction of HRP with DAB. Photomicrographs were obtained at 20× magnification, and the scale bar indicates 50 μM. Sections of representative examples of Grades I-IV astrocytoma are shown.

**Figure 6:**
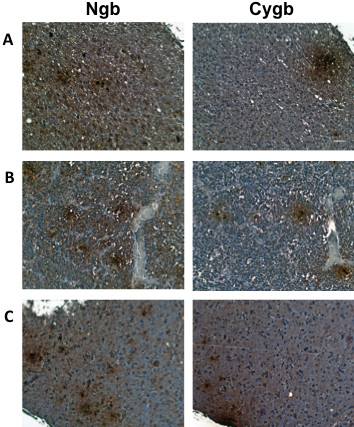
**Expression of Cygb and Ngb in human primary brain tumors**. Tissue microarrays containing cores obtained from various human brain tumors were stained with to antibodies to Ngb or Cygb. Positive staining was visualized by the chromogenic reaction of HRP with DAB. Photomicrographs were obtained at 20× magnification, and the scale bar indicates 50 μM. (A) oligodendroglioma; (B) ependymoblastoma; (C) ganglioglioma.

Both Cygb and Ngb were detected in all human brain tumors, including grades I-IV astrocytomas (Figure 5), and ependymoblastomas, gangliogliomas and oligodendrogliomas (Figure 6). Among low grade astrocytomas (Grades I and II), Ngb staining intensity was significantly greater than that of Cygb (p < 0.05). Grade III astrocytomas and GBMs also showed relatively greater Ngb staining but these differences were not significant (Tables 2 and 3). In tumors that showed distinct patterns of Ngb and Cygb expression, a comparison of tissue sections showed that these proteins were expressed in the same regions of the tumor. Large, positively staining Ngb and Cygb foci were commonly observed in GBM tumors. Approximately 60% of the GBM sections were also positive for CA IX staining which was confined to regions of the tumor also positive for Cygb and Ngb.

**Table 2:** Ngb, Cygb and CA IX expression in tissue microarrays of human primary brain tumors

Pathology	Grade	Ngb	Cygb	CA IX
Astrocytoma of right temporal lobe	II	2	1	0
Ganglioglioma of right frontal lobe	-	3	1	0
Astrocytoma of left temporal lobe	II	3	1	0
Astrocytoma of right frontal lobe	I	2	2	0
Astrocytoma	II	1	2	0
Astrocytoma	I	3	2	0
Astrocytoma of frontal lobe	I	3	1	0
Astrocytoma	I	3	1	0
Astrocytoma of right temporal lobe	II	2	1	0
Astrocytoma of brain	I	2	1	0
Astrocytoma of left occipital parietal lobe	II	2	3	0
Astrocytoma of right occipital lobe	II	3	2	0
Ganglioglioma of left temporoparietal lobe	-	3	2	1
Astrocytoma of fourth ventricle	II	3	1	1
Astrocytoma of left temporal lobe	II	5	2	0
Astrocytoma	II	5	2	0
Astrocytoma of left parietal lobe	III	3	2	0
Colloid cell hyperplasia of left brain	-	5	3	0
Astrocytoma of right temporoparietal lobe	III	4	2	0
Astrocytoma of frontal lobe	III	3	1	0
Astrocytoma of foramen magnum	III	1	0	2
Astrocytoma of left temporal lobe	II	3	2	0
Astrocytoma	II	3	2	0
Brain tissue	-	3	4	1
Astrocytoma of right temporal lobe	II	3	2	0
Astrocytoma of fourth ventricle	II	3	1	0
Astrocytoma of right temporal lobe	II	3	1	0
Astrocytoma	III	2	3	0
Astrocytoma of left temporoparietal lobe	II	3	2	0
Astrocytoma of right cerebral ventricle	II	4	3	0
Oligodendroglioma of right frontal lobe	-	4	4	0
Astrocytoma of brain	III	4	2	0
Astrocytoma of brain	III	3	4	0
Astrocytoma of left temporal lobe	II	3	2	0
Astrocytoma of left temporal lobe	II	2	2	0
Astrocytoma of intracranial	II	3	2	0
Astrocytoma of cerebellum	III	1	1	0
Astrocytoma of left temporal lobe	III	1	1	0
Astrocytoma with necrosis of left frontal lobe	III	3	2	1
Astrocytoma of left temporal lobe	III	3	1	0
Astrocytoma of temporoparietal lobe	III	4	2	0
Astrocytoma of right cerebelli	III	2	2	0
Astrocytoma	III	3	2	0
Astrocytoma	III	4	4	0
Brain tissue with colloid cell hyperplasia of right temporal lobe	-	4	*NE*	0
Astrocytoma	III	2	2	0
Astrocytoma of brain	III	2	2	0
Astrocytoma of left frontal lobe	III	3	1	0
Astrocytoma of brain	II	4	3	0
Astrocytoma of left frontal lobe	III	2	1	0
Astrocytoma of left temporal lobe	II	4	4	1
Astrocytoma of left occipital	III	3	3	0
Astrocytoma of left temporal lobe	III	4	4	0
Astrocytoma of right temporal lobe	IV	3	4	0
Astrocytoma of left temporal lobe	III	2	1	0
Astrocytoma of right temporal lobe	III	1	1	0
Astrocytoma of right temporal lobe	III	3	1	0
Astrocytoma of fourth ventricle	IV	3	2	0
Astrocytoma	II	3	3	0
Astrocytoma of temporal lobe	IV	4	2	0
Tumor necrosis tissue of right frontal region	-	4	5	1
Astrocytoma of left occipital parietal lobe	III	4	5	1
Malignant oligodendroglioma	-	2	2	0
Desmoplastic astrocytoma of left temporoparietal lobe	-	3	5	0
Astrocytoma of intracranial	II	3	2	0
Ependymoblastoma of posterior fossa	-	3	2	0
Ependymoma of brain parietooccipital	-	3	3	0
Ependymoma of brain	-	3	2	0
Ependymoma of right parietal lobe	-	4	4	0
Ependymoma of intracranial	-	3	3	0
Matched cerebrum tissue of 1st tumor	-	2	2	0
Matched cerebrum tissue of 2nd tumor	-	3	3	0
Matched cerebrum tissue of 3rd tumor	-	4	4	1
Matched cerebrum tissue of 4th tumor	-	1	2	0
Normal cerebrum tissue	-	1	2	0
Normal cerebrum tissue	-	1	1	0
Normal cerebrum tissue	-	5	2	0
Normal cerebrum tissue	-	2	3	0
Normal cerebrum tissue	-	3	4	0
Normal cerebellum tissue	-	3	1	0

Tissue microarrays constructed from cores obtained from various human primary brain cancers and normal adjacent brain were purchased from IMGENEX Corporation (San Diego, CA). Immunopositive staining for Ngb, Cygb and CA IX was assessed by two observers who noted the overall tissue staining and the presence of positively stained focal regions. Each section was assigned an overall score, where 0 = absence of positively stained cells; 1 = a few positive cells/regions ( < 10%); 2 = weak positive staining, occasional positive foci; 3 = intermediate staining, occasional positive foci ( < 50%); 4 = strongly positive in most of the section, several positive foci; and 5 = strongly positive throughout with many intensely

**Table 3:** Assessment of Cygb, Ngb and CA IX expression in tissue microarrays of human glioblastoma multiforme tumors

Pathology	Grade	Ngb	Cygb	CA IX
Glioblastoma	IV	3	1	1
Glioblastoma	IV	3	1	1
Pleomorphic glioblastoma	IV	5	4	1
Pleomorphic glioblastoma	IV	5	4	1
Glioblastoma	IV	3	1	2
Glioblastoma	IV	2	1	1
Pleomorphic glioblastoma	IV	5	2	2
Pleomorphic glioblastoma	IV	5	2	2
Glioblastoma	IV	2	2	1
Glioblastoma	IV	2	2	1
Glioblastoma (sparse)	IV	2	2	0
Glioblastoma (necrotic tissue)	IV	4	5	1
Glioblastoma (necrotic tissue)	IV	4	5	1
Pleomorphic glioblastoma	IV	4	3	0
Pleomorphic glioblastoma	IV	4	3	0
Glioblastoma	III	2	2	0
Glioblastoma	III	2	2	0
Glioblastoma	IV	2	3	1
Glioblastoma	IV	3	2	1
Glioblastoma	IV	3	2	1
Glioblastoma	III	5	4	0
Glioblastoma	III	5	4	0
Glioblastoma	IV	5	2	1
Glioblastoma	IV	5	2	2
Glioblastoma	IV	4	2	0
Glioblastoma	IV	5	2	1
Glioblastoma	IV	5	2	3
Glioblastoma	IV	3	2	0
Glioblastoma	IV	3	3	1
Glioblastoma	IV	4	3	0
Glioblastoma	IV	4	2	2
Glioblastoma	IV	4	2	0
Glioblastoma	IV	3	2	0
Glioblastoma	III	5	2	0
Glioblastoma	III	5	3	1
Glioblastoma	IV	4	2	3
Glioblastoma	IV	4	2	0
Glioblastoma	IV	5	2	0
Pleomorphic glioblastoma	IV	4	2	1
Pleomorphic glioblastoma	IV	4	3	2
Glioblastoma	III	4	3	2
Glioblastoma	III	4	3	2
Glioblastoma	III	4	2	0
Glioblastoma	III	4	3	0
Glioblastoma	IV	4	4	0
Glioblastoma	IV	5	3	0
Glioblastoma	IV	4	3	0
Pleomorphic glioblastoma	IV	5	4	1
Pleomorphic glioblastoma	IV	5	4	1
Glioblastoma	III	3	2	0
Glioblastoma	III	3	2	0
Glioblastoma	IV	4	3	3
Glioblastoma	IV	4	3	3
Glioblastoma	IV	4	4	0
Glioblastoma	IV	3	2	0
Glioblastoma	IV	4	3	0
Glioblastoma	IV	3	4	0
Glioblastoma	IV	3	4	0
Glioblastoma	IV	2	3	0
Glioblastoma	IV	3	2	0
Adjacent normal brain	-	4	3	0
Adjacent normal brain	-	3	2	1
Adjacent normal brain	-	3	4	0

Tissue microarrays constructed from cores obtained from various human glioblastoma miltiforme tumors and normal adjacent brain were purchased from IMGENEX Corporation (San Diego, CA). Immunopositive staining for Ngb, Cygb and CA IX was assessed by two observers who noted the overall tissue staining and the presence of positively stained focal regions. Each section was assigned an overall score, where 0 = absence of positively stained cells; 1 = a few positive cells/regions ( < 10%); 2 = weak positive staining, occasional positive foci; 3 = intermediate staining, occasional positive foci ( < 50%); 4 = strongly positive in most of the section, several positive foci; and 5 = strongly positive throughout with many intensely positive foci. *NE *= not evaluable.

## Discussion

Cygb mRNA and protein were detected in five human GBM cell lines cultured under aerobic conditions. However, basal Cygb protein levels varied ~19-fold. The rank-order of Cygb protein expression in these cell lines is the reverse order of that of Ngb [38], with hypoxia-tolerant cells having highest Cygb, but lowest Ngb, levels. We are not aware of any other comparisons of Cygb/Ngb concentrations in human tumor cell lines. While our results in GBM cell lines hints at a reciprocal relationship between Cgyb/Ngb, at least in GBM cell lines, this would have to be confirmed in a larger study. Co-expression of Ngb and Cygb has been reported among various structures of the anterior segment of human and canine eyes, including the cornea, iris, iridocorneal angle and the cilliary body [40], and in human retinal neurons and pigmented epithelium [41]. However, the relative expression of each protein within specific cellular structures was not quantified. In the murine retina, divergent expression of Cygb and Ngb has been reported, with Ngb levels being significantly higher [42].

Both Cygb transcript and protein were significantly increased in four of five GBM cell lines cultured under conditions that simulate *in vivo *O_2 _concentrations found in the hypoxic regions of human tumors [43,44]. Others have also shown that the hypoxia up-regulates Cygb transcript and protein in cell lines of neuronal origin-- HN33 [18] and (SH-SY5) [45]. Hypoxia-tolerant M006x cells that express high basal level of Cygb protein showed a small, but non-significant, increase in Cygb after exposure to reduced [O_2_]. Nonetheless, M006x cells are hypoxia-responsive as we have shown that expression of another hypoxia-inducible gene, *VEGF*, can be up-regulated at equivalent levels of hypoxia [46]. It is interesting to note that while Cygb levels varied widely among GBM cells under aerobic condition (19-fold), when exposed to hypoxia, the differences among the cell lines were notably reduced. This would suggest that mechanisms exist to allow cells to titrate intracellular concentrations of Cygb to optimally meet the demands of given levels of oxidative stress.

In addition to GBM cell lines, Cygb expression was also observed in both low and high grade human astrocytomas, including GBM. To our knowledge, this is the first report of Cygb expression in human primary brain cancers although high Cygb levels have been reported in brain metastases of two patients with alveolar soft part sarcomas [47]. Both Cygb transcript and protein have been reported in normal neurons, but not glia [41]. To date, there is little information concerning the co-expression of these two globin molecules in malignant human tissues. In this study, Ngb protein levels in Grades I and II astrocytomas were significantly greater than Cygb levels (p < 0.05). There was also a trend toward increased Cygb expression as tumor grade increased from I - IV, however, this would have to be confirmed in a larger study. A similar pattern of greater Ngb/Cygb positivity was also observed among non-brain cancers specimens examined, with Ngb expression significantly higher than that of Cygb (p <0.001). Regions of necrosis are a pathognomonic feature of GBM tumors, and in many sections, enhanced Cygb/Ngb staining was observed adjacent to necrotic tissue. The microenvironment of these perinecrotic regions is likely to be severely hypoxic and thus, hypoxia-inducible proteins would be expected to be upregulated. In order to test this, we used CA IX staining as an endogenous marker of tumor hypoxia. *CA IX *is a target gene of the hypoxia-inducible transcription factor, HIF-1α, and some, but not all, studies have shown a correlation between CA IX expression and tumor hypoxia (reviewed in [48]). In these TMAs, tumor regions that were positive for CA IX also showed Cygb/Ngb expression. However, not all regions that were Ngb/Cygb positive also showed CA IX immunoreactivity. A possible explanation is the fact that in tumors, CA IX expression is generally associated with perinecrotic regions that are likely to be severely hypoxic [48]. In contrast, the up-regulation of both Ngb and Cygb expression has been observed at relatively higher O_2 _tensions (~1% O_2_).

In addition to the brain tumor TMAs, we also studied Ngb and Cygb expression in several normal tissues and their malignant counterparts. Although Cygb has been described as a ubiquitous cellular globin, it was reduced or absent from many normal tissues, but prominent in ductal cells in gallbladder, stomach, small bowel and kidney. Shigematsu *et al. *[49] previously reported a similar distribution for Cgyb in normal tissues. However, the most striking feature observed in our study was the distinct regional co-localization of Ngb, Cygb and CA IX in several normal tissues, including stomach, small bowel and salivary gland. CA IX expression in human alimentary tract has been previously described [50], but to our knowledge, this is the first demonstration of co-expression of Ngb/Cygb/CA IX in human tissue.

To corroborate these protein expression patterns, we queried NCBI Gene Expression Omnibus (GEO) Profiles--a public repository of microarray gene expression data [51]. This search indicated that there is robust expression of Ngb and Cygb mRNA in a variety of human primary non-malignant and tumor samples [See Additional files 1 and 2]. However, there is considerable heterogeneity in the expression Ngb and Cygb mRNA, both *within *a given histological type of non-malignant or tumor tissue, and *among *different types of tissue.

Although Ngb and Cygb were discovered almost a decade ago, their functions continue to be explored. Currently, evidence from both *in vitro *and *in vivo *model systems indicates that these globins play important roles in protecting cells from death during ischemic insult (hypoxia-reperfusion), oxygen/glucose deprivation, or exogenous oxidative stress [17,22,45,52-55]. Recently, Raychaudhuri *et al. *[56] used experimental and computational analyses to show that Ngb overexpression protects SH-SY5Y neuroblastoma cells from cell death by inhibiting the initiation of the intrinsic apoptotic pathway. They hypothesize that the ability of Ngb to bind to cytochrome *c *provides a mechanism whereby Ngb could interfere with cytochrome *c*-induced activation caspase-9, a critical step required for initiation of the intrinsic apoptotic cascade. Accordingly, Ngb or Cygb expression may provide the same pro-survival effect in tumors in which both chronic and intermittent hypoxia (ischemia/reperfusion) as well as glucose deprivation commonly occur (reviewed in [33].

## Conclusions

We showed two novel globins--Cygb and Ngb, are expressed in human primary tumors and cell lines. Their up-regulation by hypoxia *in vitro*, and their association with an endogenous hypoxia marker in tumor tissue sections suggests that these globins may contribute to the pro-survival mechanisms utilized by these cancers to survive under conditions of oxidative stress.

## Methods

### Cell lines and in vitro culture conditions

The origin and characterization of the GBM cell lines have been published previously: the M059J (ATCC number CRL2366) and M010b cell lines are hypoxia-sensitive; the M006x and M006xLo cell lines are hypoxia-tolerant [46,57,58]. The U87T cell line was established following serial *in vivo *selection in a glioma mouse model [59] and was kindly provided by Dr. Donna Senger (University of Calgary). Its relative sensitivity to hypoxia has not been determined. All cells were maintained as monolayer cultures in DMEM/F12 media supplemented with 10% fetal calf serum and 1 mM L-glutamine in a humidified atmosphere of 5% CO_2 _in air at 37°C. All tissue culture supplies were purchased from GIBCO.

### Generation of hypoxia in vitro

A de-gassing manifold was used to generate hypoxia *in vitro *[60]. Exponential phase cells (~2 × 10^5^) were seeded onto 60-mm glass plates and then incubated under standard laboratory culture conditions (5% CO_2 _in air) for 4 days. The medium was then replenished and the plates were transferred to aluminum chambers from which the air was evacuated and then replaced with 5% CO_2_/balance N_2 _until an O_2 _tension of 0.6% was achieved. The sealed, air-tight aluminum chambers were then incubated at 37°C for 6-48 h. At the end of each incubation interval, the aluminum chambers were unsealed, the tissue culture plates removed, RNA and protein isolated from the cells as described in Section 2.4 and 2.5.

### Quantitative real-time reverse transcription-PCR

Total RNA from cultured cell lines was isolated using the RNeasy mini kit (QIAGEN). Reverse transcription (RT) was carried out with 1 μg total RNA. per 20 ml reaction volume using QuantiTect reverse transcription kit (QIAGEN). RT experiments were performed with GeneAmp PCR system 9700 (Applied Biosystems). Quantitative real-time PCR (qRT-PCR) analysis was performed with a 7900 HT Fast Real-Time PCR System (Applied Biosystems) using TaqMan fast universal PCR master mix and a validated TaqMan Gene Expression assay (Applied Biosystems) for the human CYGB gene (assay ID: Hs00370478_ml). Human 18 S rRNA gene (part no.: 4333760T, Applied Biosystems) was used as endogenous control. Amplification data were analyzed with SDS RQ Manager 1.2. Relative quantities of Cygb transcripts were normalized against relative quantities of the 18 S rRNA transcripts, and fold-expression changes calculated using the expression 2^-ΔΔCT^.

### Western blotting

Whole-cell lysates were prepared using complete Lysis-M buffer (Roche Diagnostics) and protein content determined using a protein assay kit (Pierce). Equal amounts of protein (50 μg) were resolved using 13% SDS -PAGE under reducing condition and electro-transferred to polyvinylidene difluoride membranes (Bio-Rad Laboratories, CA). Membranes were blocked with 5% skim milk then incubated with Cygb antibody (1:60 rabbit anti-human polyclonal antibody [sc-66855] Santa Cruz Biotechnology, Inc, CA) or α-tubulin antibody (1:5000, mouse anti-human α-tubulin monoclonal antibody, SIGMA) as loading control. Membranes were incubated with polyclonal goat anti-rabbit IgG (H+L) horseradish peroxidase (HRP)-conjugated (1:25000, Jackson ImmunoResearch Laboratories, Inc. PA) or polyclonal goat anti-mouse IgG HRP-conjugated (1:6000, DakoCytomation Denmark A/S, Demark). Bound proteins were detected using chemiluminescence reagents (SuperSignal West Pico Chemiluminescent Substrate, Thermo Scientific, IL) and visualized by exposing to X-ray film (Fuji Photo Film, Japan) that was developed using a Kodak X-OMAT 2000A processor (Eastman Kodak Company, Japan). X-ray films were scanned using an Artixscan 1800f scanner (MICROTEK, TAIWAN) and bands were analyzed using Quantity One 1-D analysis software (Bio-Rad Laboratories). The integrated areas of bands were determined and expressed in arbitrary units (AU).

### Tissue micorarrays and immunostaining

Tissue microarrays (TMA) constructed from tissue sections obtained from various human solid tumors and normal adjacent tissues (IMH-346), GBMs (IMT-01256), and various brain cancers and normal adjacent tissues (IMT-01257) were purchased from IMGENEX Corporation (San Diego, CA). The manufacturer confirmed the glial origin of the brain tumors included on the arrays using immunoreactivity to anti-glial fibrillary acid protein. Immunohistochemical staining was performed according to manufacturer's protocol with modifications. TMA slides were deparaffinized by incubating in a dry oven at 62°C for 1.5 h, dewaxed in xylene, and then hydrated. TMAs were processed for antigen retrieval, incubated with blocking buffer (0.5 M glycine in PBS-0.2% brij35) and then with antibodies to Ngb (1:150, mouse anti-human Ngb monoclonal antibody, BioVendor Laboratory Medicine Inc., Czech Republic), Cygb (1:150, mouse anti-human Cygb monoclonal antibody, Abnova Corporation, Taiwan) or Ca IX (1:600, rabbit anti-human CA IX, Novus Biologicals, Inc., USA). The specificity of the primary antibodies was demonstrated by using Cygb or Ngb peptides to block positive immunostaining [Additional file 3]. Tissue endogenous peroxidase was quenched by incubation with 3% H_2_O_2 _for 15 min. Tissue sections were incubated with HRP-conjugated goat anti-mouse antibodies (DakoCytomation) and positive staining visualized by the chromogenic reaction of HRP with DAB (3,3' diaminobenzidine tetrahydrochloride) (DakoCytomation). Relative Cygb and Ngb expression in each tissue core was assessed by two observers who noted the overall tissue staining and the presence of positively stained focal regions. Each section was assigned an overall score, where 0 = absence of positively stained cells; 1 = a few positive cells/regions (< 10%); 2 = weak positive staining, occasional positive foci; 3 = intermediate staining, occasional positive foci (< 50%); 4 = strongly positive in most of the section, several positive foci; and 5 = strongly positive throughout with many intensely positive foci.

### Statistics

Data from four replicate experiments were expressed as mean ± standard error. Statistical analyses were performed using SigmaPlot 11 software (Systat Software, Inc). Differences between groups were compared using one-way ANOVA or ANOVA on ranks (Kruskal-Wallis) based on the normality and equal variance tests. To determine exactly which groups are different and the size of the difference, multiple comparisons versus control group were carried out using Bonferroni t-test and Dunnett's or Dunn's test for one-way ANOVA and ANOVA on ranks (Kruskal- Wallis), respectively, as post-hoc tests.

## Competing interests

The authors declare that they have no competing interests.

## Authors' contributions

EM and JAT conceived of the study. EM performed the *in vitro *analyses and TMA immunostaining, did the statistical analyses and wrote the first draft of the manuscript. ART and JAT analyzed the TMAs. All authors contributed to the final draft of the manuscript.

## Supplementary Material

Additional file 1 **Table S1. Results of GEO profiles query of Cygb expression in human non-malignant and tumor tissues**. This table provides the range of the intra-sample percentile rank values for Cygb expression in a variety of human primary tissue samples. These values provide an indication of the relative expression of Cygb compared to that of all other genes expressed in that sample. Click here for file

Additional file 2 **Table S2. Results of GEO profiles query of Ngb expression in human non-malignant and tumor tissues**. This table provides the range of the intra-sample percentile rank values for Ngb expression in a variety of human primary tissue samples. These values provide an indication of the relative expression of Ngb compared to that of all other genes expressed in that sample. Click here for file

Additional file 3 **Figure S1. Antibody specificity demonstrated by competitive immunostaining**. This figure shows that incubation of primary Ngb and Cygb antibodies with the relevant recombinant proteins effectively blocked positive immunostaining. Click here for file
