# Supplementary figures and images for: Hypoxic regulation of cytoglobin and neuroglobin expression in human normal and tumor tissues
Source: Cancer Cell Int. 2010 Sep 9;10:33. doi: 10.1186/1475-2867-10-33 (PMC2945342; doi:10.1186/1475-2867-10-33)

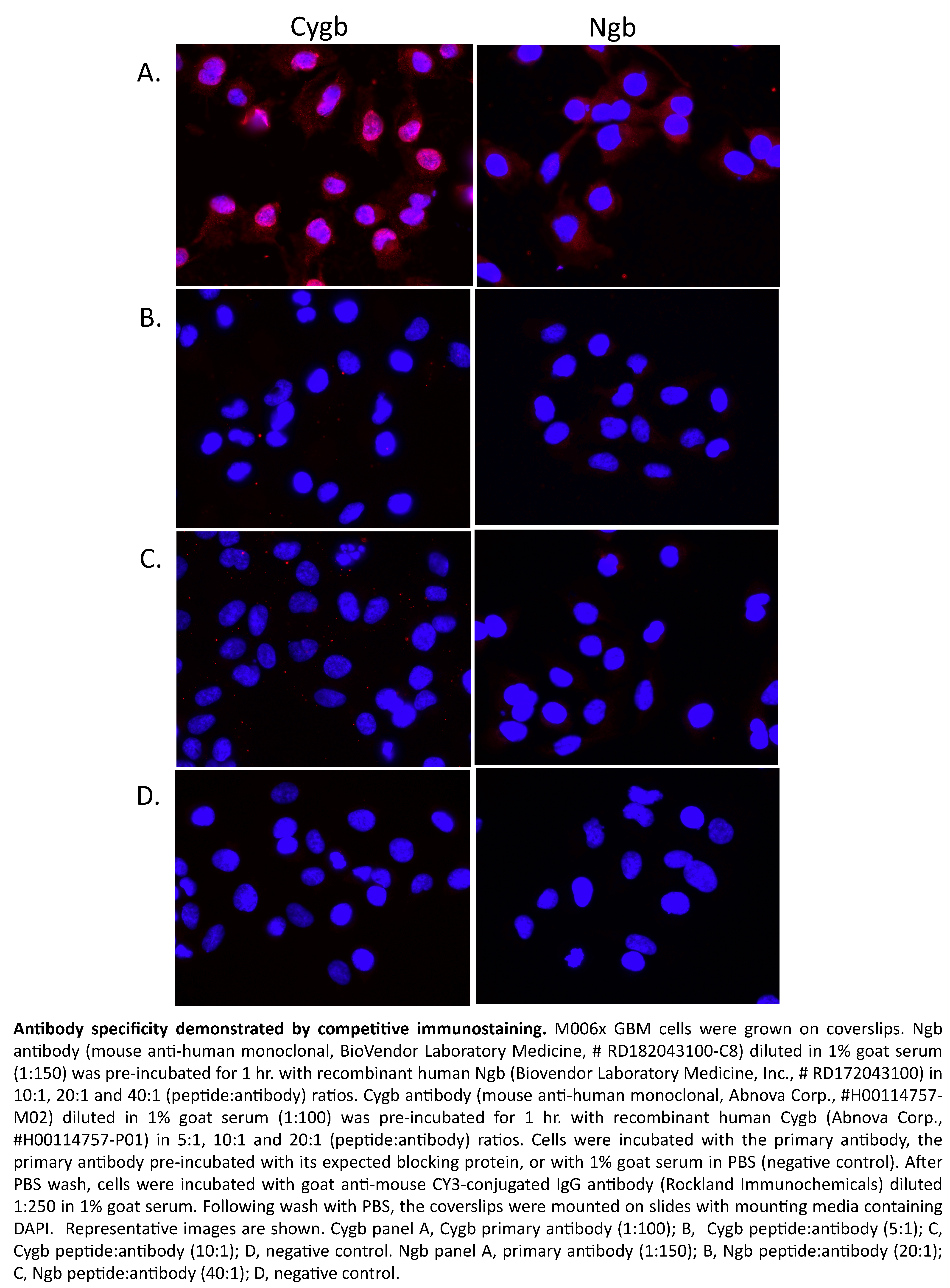

Supplement: Additional file 3 — Figure S1. Antibody specificity demonstrated by competitive immunostaining. This figure shows that incubation of primary Ngb and Cygb antibodies with the relevant recombinant proteins effectively blocked positive immunostaining. [file 1475-2867-10-33-S3.PNG]
